# A Case of Pericardial Decompression Syndrome Following Surgical Pericardial Fluid Drainage

**DOI:** 10.7759/cureus.16631

**Published:** 2021-07-26

**Authors:** John Abdelmalek, Mostafa M Abohelwa, Mohamed Elmassry, Mohammad M Ansari

**Affiliations:** 1 Internal Medicine, Texas Tech University Health Sciences Center, Lubbock, USA; 2 Cardiology, Texas Tech University Health Sciences Center, Lubbock, USA

**Keywords:** pericardial decompression syndrome, pds, cardiac tamponade, pericarditomy, pericardiocentesis

## Abstract

Pericardial decompression syndrome (PDS) is a rare and serious complication that follows often-initially-uncomplicated pericardial drainage in patients with pericardial effusion and tamponade physiology. The pathophysiology of PDS is not yet completely understood, although several mechanisms have been postulated. In this report, we present a case of PDS in a 70-year-old male with end-stage renal disease (ESRD) after he underwent a surgical pericardial window for drainage of a moderate pericardial effusion with tamponade physiology. This case provides further evidence that rapid pericardial decompression, notably with pericardiotomy, can lead to acute life-threatening low cardiac output heart failure, particularly in patients with underlying cardiac risk factors. Early recognition, diagnosis, and supportive treatment in the ICU are crucial for improving survival rates in these patients.

## Introduction

Cardiac tamponade is a medical emergency that is caused by fluid accumulation in the pericardial space. When fluid accumulates rapidly or excessively, it can lead to inappropriate filling of cardiac chambers, ultimately resulting in impaired cardiac hemodynamics, hypotension, and eventually cardiac arrest [1]. Often underreported, pericardial decompression syndrome (PDS) is characterized by the paradoxical deterioration of cardiac hemodynamics and the development of acute low cardiac output heart failure and pulmonary edema after uncomplicated pericardial drainage [2].

Several plausible mechanisms have been put forward to explain the etiology of this syndrome, incorporating hemodynamic effects of a sudden increase in venous return following fluid drainage, acute myocardial stunning, and acute withdrawal of sympathetic overdrive. PDS incidences are very rare and often go underreported. Hence, close hemodynamic monitoring and judicious pericardial fluid drainage in patients undergoing either pericardiocentesis or surgical pericardiectomy are of paramount importance.

## Case presentation

A 70-year-old male with a significant past medical history of diabetes, hypertension, hyperlipidemia, and end-stage renal disease (ESRD) on regular hemodialysis presented to our facility's emergency department after he had been found to have significant dyspnea, hypoxia, and hypotension at the outpatient pulmonary clinic. His chest X-ray showed stable bilateral chronic pleural effusions and a significant increase in cardiac silhouette. Transthoracic echocardiogram (TTE) in the ER showed moderate pericardial effusion with echocardiographic evidence of tamponade physiology. His left ventricular ejection fraction (EF) was found to be 50-54%.

The patient underwent an uncomplicated emergent surgical pericardiotomy with a pericardial window; 400 ccs of serosanguineous effusion were drained and he was sent to the ICU unit for postoperative recovery and close monitoring with a pericardial drain in place. His vital signs initially improved after the drainage of pericardial fluid, with normalization of blood pressure and reduced oxygen requirements from 5 L to 2 L.

However, a few hours after the surgery, the patient started to develop pulmonary edema and respiratory acidosis, requiring noninvasive positive pressure ventilation; his blood pressure significantly dropped, necessitating escalating vasopressor support. The patient was later intubated and mechanically ventilated for impending respiratory failure. Notably, a previous TTE done two months before this hospital admission had shown a normal left ventricular systolic function (EF estimated at 70%) and preserved right ventricular systolic function as well with only trace pericardial effusion.

**Figure 1:**
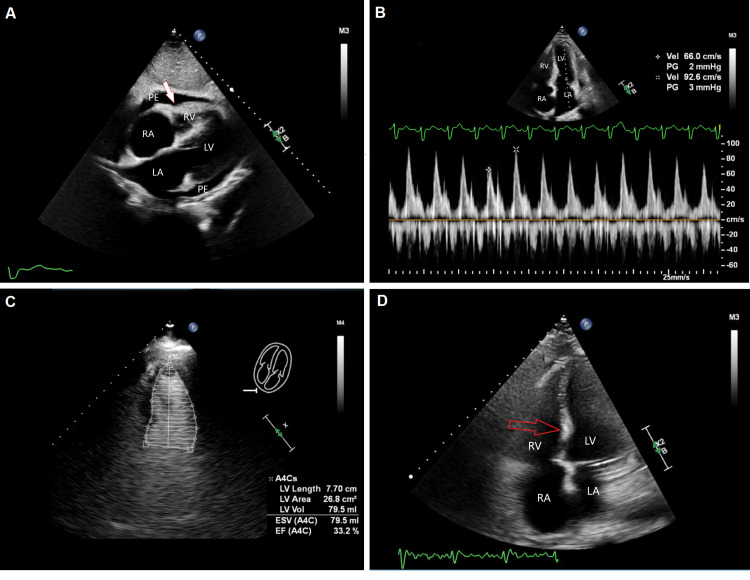
Transthoracic echocardiography A: subxiphoid four-chamber view of the heart demonstrates moderate PE with evidence of early right ventricular end-diastolic compression (marked by white arrow). B: apical four-chamber view pulsed-wave Doppler of mitral valve inflow shows significant variability as an early sign of tamponade physiology. C: after surgical pericardiotomy: contrast echocardiography of left ventricle in the apical four-chamber view shows significantly decreased ejection fraction estimated at 33.2%. D: after surgical pericardiotomy: apical four-chamber view shows (foreshortened) right ventricle with a paradoxical septal shift towards the left (marked by red arrow) RV: right ventricle; LV: left ventricle; RA: right atrium; LA: left atrium; PE: pericardial effusion

TTE done in the ICU postoperatively showed a new finding of moderately reduced left ventricular EF of 30-34%, with moderate global myocardial wall hypokinesis more pronounced at apical, lateral, and inferior walls; right ventricular systolic function was significantly reduced as well [tricuspid annular plane systolic excursion (TAPSE): 0.9] (Figure 1).

EKG showed no acute ischemic changes; cardiac biomarkers were elevated at 155 ng/L, and trended up to 156 ng/L but with no significant serial rise in the setting of ESRD (flat curve). A diagnosis of PDS was made. Acute pulmonary embolism (PE) was excluded by Wells criteria, absence of deep vein thrombosis (DVT) by venous duplex, as well as atypical echocardiographic features for massive PE in this patient. Sepsis workup was negative as well, and hemopericardium/myocardial puncture was excluded.

Supportive treatment in the ICU unit was continued, and vasopressors were weaned off gradually over a period of three days. The patient was successfully extubated, and his fluid status improved with continuous renal replacement therapy. The pericardial drain was removed after 72 hours as well; another 200 mL of serosanguineous fluid was drained before it was removed. An echocardiogram done after the patient hemodynamically stabilized showed improved left ventricular systolic function with EF estimated at 45-49%; inferior wall hypokinesis had improved with remaining mild lateral and apical walls hypokinesis. He was transferred to the stepdown unit and later discharged after 13 days of admission.

A follow-up TTE six months after discharge showed normalization of his left ventricular systolic function with EF estimated at 50-54%, and no significant wall motion abnormalities were observed.

## Discussion

Pericardial decompression syndrome is a term that was first proposed by Angouras et al. in 2010 [2]. However, the first described case actually dates back to 1983 when Vandyke et al. reported pulmonary edema in a 42-year-old male patient with malignant pericardial effusion after undergoing pericardial drainage [3]. It is also known as post-pericardial drainage low cardiac output syndrome. It is estimated that the incidence of PDS is less than 5%, which goes up to 34% following drainage of malignant pericardial effusion [4,5]. There is no single explanation regarding the pathophysiology of PDS [6]. Multiple theories have been proposed to explain the paradoxical hemodynamic instability after either needle or surgical decompression of cardiac tamponade. This can lead to cardiogenic shock, pulmonary edema, and even death [1,7]. Paradoxical worsening of the hemodynamics following pericardial fluid drainage can also ensue in rare cases, resulting in PDS.

Vandyke et al. have proposed that preload/afterload mismatch is the mechanism behind pulmonary edema and PDS development [3]. Rapidly increased venous return following pericardial fluid drainage leads to increased right ventricle expansion at the expense of left ventricle filling (preload) and therefore afterload, resulting in pulmonary edema that is even exacerbated by persistently increased left ventricular afterload due to adaptive sympathetic/neurohormonal increase in systemic vascular resistance activated by the low cardiac output physiology in tamponade [4,8,9].

In addition to the above-mentioned hemodynamic theory, Wolfe and Edelman have reported that pericardial decompression and deactivation of the adaptive autonomic/sympathetic stress response with tamponade might unmask an underlying myocardial dysfunction, and, interestingly, can lead to new-onset (de novo) transiently impaired systolic function similar to that in patients with Takotsubo cardiomyopathy [9]. Lastly, Skalidis et al. have demonstrated that the increased pericardial pressure in tamponade can limit coronary vascular perfusion [10], resulting in myocardial stunning. This can result in significant left ventricular dysfunction, magnifying the PDS risk.

Patients who develop PDS usually present with left ventricular failure with or without shock and pulmonary edema; isolated right ventricular failure and biventricular failure have also been reported. The onset of symptoms can be immediate or delayed up to 48 hours after pericardial fluid drainage [11]. Other causes of shock should be excluded before establishing the diagnosis of PDS.

While treatment is usually supportive and focused on the management of cardiogenic shock in a critical care unit [6,11], prevention should be the preferred strategy. Several risk factors for the development of PDS have been described in the literature. Rapid and large-volume pericardial fluid drainage can lead to the development of PDS [11-13]. Hence, it has been observed more with surgical pericardial drainage [14]. Malignant pericardial effusion is another known risk factor. Other conditions such as the presence of pericardial calcifications, prior radiation therapy, pre-existing cardiomyopathy with low EF, and connective tissue disorders have also been linked to PDS in multiple case reports [15].

Small volume, staged pericardial fluid drainage (through an indwelling pericardial drain catheter) is often recommended so that PDS can be prevented. The initial amount of fluid removed should be guided by the clinical and echocardiographic improvement of tamponade physiology [4,13]. However, PDS has been documented even after small volume pericardial drainage [16]. Special consideration should be given to patients with malignant pericardial effusion as well as the above-mentioned risk factors.

In our case, we suspect that the patient developed transient biventricular failure and subsequent cardiogenic shock with pulmonary edema secondary to preload/afterload mismatch. This was precipitated by significantly increased venous return after a sudden release of intrapericardial pressure and persistently increased afterload due to sympathetic overdrive. Sympathetic stress response might have also precipitated myocardial stunning as evidenced by significant myocardial hypokinesis seen in the patient’s echocardiogram.

## Conclusions

PDS is an underreported, potentially fatal complication in cardiac tamponade patients who undergo pericardial fluid drainage. Our report is the first one to document the development of PDS following a relatively small volume (400 ml) of pericardial window drainage of pericardial effusion associated with tamponade. Physicians’ familiarity with the syndrome, risk factors, and clinical presentation are crucial to establishing an early diagnosis and providing prompt treatment in a critical care setting.
